# Design and Construction of a Chamber Enabling the Observation of Living Cells in the Field of a Constant Magnetic Force

**DOI:** 10.3390/cells10123339

**Published:** 2021-11-28

**Authors:** Daniel Dziob, Jakub Ramian, Jan Ramian, Bartosz Lisowski, Jadwiga Laska

**Affiliations:** 1 Faculty of Pharmacy, Jagiellonian University Medical College, Medyczna 9, 30-688 Kraków, Poland; 2 Faculty of Mechanical engineering and Robotics, AGH University of Science and Technology, Al. Mickiewicza 30, 30-059 Kraków, Poland; ramian@student.agh.edu.pl; 3 Faculty of Medical Sciences in Katowice, Medical University of Silesia, Poniatowskiego 15, 40-055 Katowice, Poland; s75638@365.sum.edu.pl; 4 Faculty of Medicine, Jagiellonian University Medical College, św. Łazarza 16, 31-530 Kraków, Poland; bartek.lisowski@uj.edu.pl; 5 Faculty of Materials Science and Ceramics, AGH University of Science and Technology, Al. Mickiewicza 30, 30-059 Kraków, Poland; jlaska@agh.edu.pl

**Keywords:** living cell observation, magnetic field, optical microscopy, Halbach magnet

## Abstract

The aim of the work was to design and construct a microscopic stage that enables the observation of biological cells in a magnetic field with a constant magnetic force. Regarding the requirements for biological observations in the magnetic field, construction was based on the standard automatic stage of an optical microscope ZEISS Axio Observer, and the main challenge was to design a set of magnets which were the source of a field in which the magnetic force was constant in the observation zone. Another challenge was to design a magnet arrangement producing a weak magnetic field to manipulate the cells without harming them. The Halbach array of magnets was constructed using permanent cubic neodymium magnets mounted on a 3D printed polymer ring. Four sets of magnets were used, differing in their dimensions, namely, 20, 15, 12, and 10 mm. The polymer rings were designed to resist magnetic forces and to keep their shape undisturbed when working under biological conditions. To check the usability of the constructs, experiments with magnetic microparticles were executed. Magnetic microparticles were placed under the microscope and their movement was observed to find the acting magnetic force.

## 1. Introduction

The influence of a magnetic field on organisms has been intensely studied since the 1970s, after the evidenced reporting of a link between human cancer and exposure to magnetic fields (MF) [1,2]. The subject is interesting in several aspects. Weak magnetic fields are under special surveillance due to their interactions with biological materials [1,3,4]. It is expected that people and other biological organisms will be exposed in the near future to magnetic fields due to the prospective space flights available to civilians [3]. It is also known that some animals use magnetic fields for navigation [5], and that MF can influence the concentration of some biomolecules [6]. Hence, magnetoreception in living organisms and magnetotactic species are currently being studied extensively [7]. The impact of the magnetic field on biological cells has been discussed both from a therapeutic point of view and because of the potential undesirable effects [8,9]. The novel subject of this study is the induced interaction of the magnetic field with cells containing magnetic nanospheres. This gives many opportunities to manipulate cells, e.g., cause quantitative separation of cells or cell migration [10,11,12].

The study of the effects of magnetic fields on biological processes is very difficult, not only because the effects are weak. The crucial problem is to adjust the techniques to investigate them live and in real time. Optical microscopy is the most widely used technique for the observation of cells, however, it is not yet adapted for the observation of living cells in a magnetic field. The first approach was carried out by Woodward and Ikeyaa who, using a custom microscope, demonstrated that flavin-based autofluorescence in native, untreated HeLa cells is magnetic field sensitive, due to the formation and selective electron spin recombination of spin-correlated radical pairs [13]. The magnetic field was supplied to the sample using a projected vector field electromagnet capable of generating a magnetic field in any arbitrary direction relative to the sample. The magnet was mounted at a distance of 5 mm or less above the sample slide to generate an appropriate field strength and to ensure the uniformity of the magnetic field. A simple mechanical system with a permanent magnet that produces a rotating magnetic field of nearly constant amplitude in the focal plane of a light microscope was designed by Smid, Shcherbakov, and Petersen [14]. The designed system allowed investigation of the magnetic and hydrodynamic properties of magnetotactic bacteria. Living bacteria exposed to electromagnetic fields were also investigated with an optical microscope. In this case, a custom device was also designed that allowed for a continuous microscopic observation of cells and their progression to mitosis before and after exposure to the magnetic field [15]. Electromagnetic field exposures were performed in a transverse electric magnetic chamber mounted on a specially designed holding frame on an inverted microscope. The chamber was placed inside an incubator chamber that covered the microscope stage to control the culture conditions during exposure. The biocompatibility of iron nanoparticles coated with a graphite shell and their localization in living tumor cells has been investigated by confocal microscopy [16].

The prospect of the creation of weak uniform magnetic fields for biological measurements has attracted the attention of researchers for a long time [17,18,19]. It is often forgotten that the magnetic field and the magnetic force are not the same physical phenomena. In a constant (homogenous) magnetic field, there is no magnetic force and particles with magnetic moment can only rotate. For the occurrence of a magnetic force, which can cause a displacement of such particles, a magnetic field gradient is necessary [20]. Therefore, to study the influence of a magnetic force, a system with a specific, preferably linear, gradient is necessary. One approach to obtain such a field was presented in the 1980s by Klaus Halbach, who invented an array of magnets to focus particle accelerator beams [21,22,23]. The array is formed from permanent bar magnets, equally magnetized, oriented, and positioned according to analytical equations, which predict the desired outcome. Our special interest was focused on the Halbach cylinder, producing a magnetic field localized entirely within the cylinder, with zero field outside. Similar gradient fields can be produced with coils, but such a system has two major drawbacks to biological research applications. First, the flowing current generates Joule’s heat, thereby raising the temperature of the sample. Second, the system of coils takes up a lot of space and often makes it impractical or impossible to be placed in the microscope. Therefore, Halbach arrangement was chosen because of the high stability of the static magnetic field, very small stray field, simplicity and compactness of the construction, and its small weight. Dogan et al. designed a modified Halbach system to be used in portable NMR equipment, which served as our inspiration [24].

The aim of our work was to design a magnet system producing the required magnetic field and enabling the observation of living organisms, which can be applied in an optical microscope. The point was to obtain magnetic fields weak enough to be safe for living cells, but at the same time strong enough to trigger the displacement of a fluorescent magnetic bead, which, for example, can serve as source of internal mechanical forces for the cells. The Halbach magnet system was elaborated and applied.

## 2. Materials and Methods

Polylactide (PLA), polycarbonate (PC), polybutadiene-graft-poly(acrylonitrile-co-styrene) (ABS) filaments were purchased from Ultimaker. Poly(ethylene terephthalate) glycol-modified (PET-G) was purchased from 3D4MAKERS.

All models were printed on an Ultimaker 3 3D printer; a semi-closed printer, which ensures a more uniform temperature of the air surrounding the object during printing than open devices. To ensure good adhesion between the glass printer base and the printout, Ultimaker adhesive mats were used. The printer was fitted with a Print Core AA 0.40 mm diameter nozzle. Ultimaker Cura version 4.6 slicer was used to convert digital 3D models into printing instructions for a printer. An adhesive mat to stick to the first layer was purchased from Ultimaker.

The chamber was designed to work with Zeiss Axio Observer 7 inverted fluorescent microscope. The models were designed in Autodesk Inventor Professional 2019 software. They were mounted on a default motorized scanning stage. The microscope was fitted with ZEISS alpha plan apochromat 63×/1.46 Oil Korr m27 oil lens. Glass bottom dishes (Cellvis D29-20-1N) were chosen for microscopic observations, since their 33 mm outer dimension made them easy to fit inside the Halbach ring arrays. Cubical neodymium magnets (edge lengths were 10, 12, 15, and 20 mm), made from N42 material [25], were used as sources of the field.

The induction of a magnetic field in the array was measured with Asonik SMS 102 sensor (TEL-Atomic Inc./Asonik, Poland). For the measurements of a magnetic force 500 nm magnetic, fluorescent microparticles with iron oxide γ-Fe_2_O_3_ core were used (screenMAG, Chemicell, Germany). Thermal tests were conducted in the Thermo Scientific series 8000 WJ CO_2_ cell incubator.

## 3. Results

### 3.1. Designing the Magnetic Chamber for the Microscopic Observation

**Figure 1:**
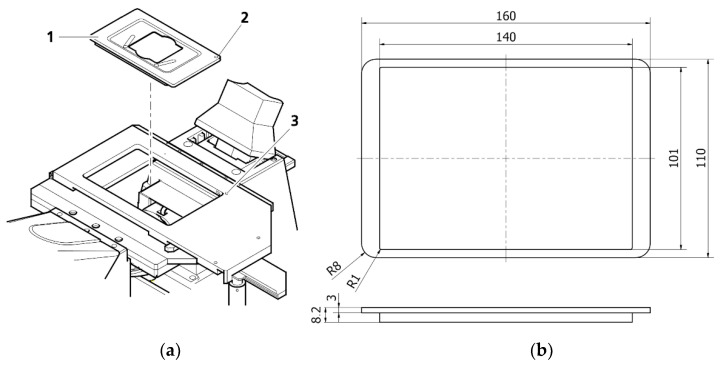
Designing the set for observation of living cells: (**a**) View of an observation area of Zeiss Axio Observer (from the Operating Manual), 1—mounting frame, 2 and 3—dots indicating the position to ease alignment.; (**b**) Geometrical restrictions for the magnetic frame in mm.

**Figure 2:**
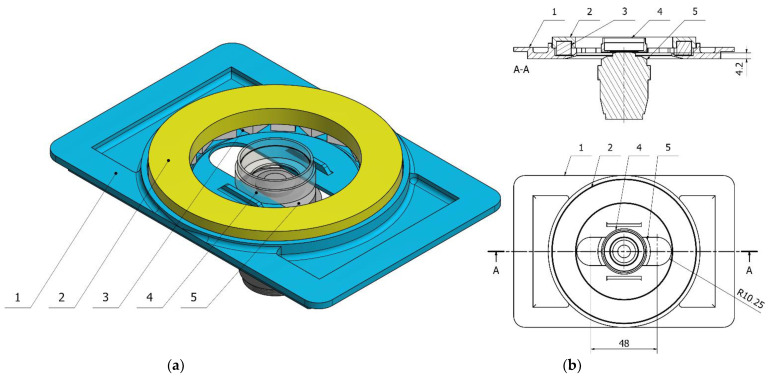
(**a**) 3D schematic diagram of the assembled Halbach magnet; (**b**) Technical drawing of the magnet assembly in cross section (upper right) and top view (lower right); 1—base, 2—ring, 3—magnet, 4—glass-bottom Petri dish, 5—microscope objective.

The Zeiss Axio Observer 7 fluorescent microscope’s architecture defined the fundamental geometrical constraints for our design. The microscope was equipped with a digitally controlled scanning stage with interchangeable default mounting frames (Figure 1). The matching frames with a set of rings were 3D printed. The ring should be mountable to the frame, transforming it into a Halbach array (Figure 2). All specimens were positioned in the axis which passes through the middle of the magnet, which maximized the resulting magnetic forces.

Halbach array of magnets allows for obtaining a quadrupole magnetic field. By summing up the fields of single permanent magnets, the magnetic induction of the resulting magnetic field increases radially from the center in the direction of the ring’s circumference. By changing the size of the magnets, one can change the resulting magnetic field.

The mounting frame not only supports the ring. It also has to support the specimen. We optimized its design to fit Petri dishes with a diameter of 20 mm. Glass bottom dishes were chosen to improve the quality of the image and allow the use of lenses with higher magnification. This is related to the focal length of each lens—the greater the magnification, the smaller the required distance between the lens and the sample. Thanks to the versatility of 3D printing, the frame can also be easily modified to support glass or plastic slides. An important aspect of the design was its ability to work with oil lenses, which require almost direct contact with the sample. They are also wider than the air lenses. The bottom of the frame was designed to allow measurements in the largest possible area inside the ring-shaped magnet array.

The main goal of the design was to allow for the observation of the movement of living cells in the magnetic field. Thus, the frame nor any other element must not be deformed during the measurement, as the deformation could affect the leveling of the slide or a dish, and this, in turn, could disturb the position determination and lead to errors in the measurement of the cell displacement. For this reason, it is essential to select the appropriate printing parameters and, in particular, the material. Biologically relevant experiments are often carried out at 37 °C and the increased humidity necessary for the cells to survive the 72 h observation. Therefore, the influence of the degree of filling in the print and the type of material were tested, as well as the influence of the magnets on the mechanical stability and integrity of the frame in operational conditions.

In summary, the proposed complete setup consists of a 3D printed mounting frame and interchangeable magnetic rings, which are mounted on the frame to obtain a constant gradient magnetic field in the microscope’s field of view. Below we describe three aspects of the setup: material considerations, magnetic properties, and simple experimental validation.

### 3.2. 3D Printing of the Frames and Mechanical Testing

Since there are no universal and unambiguous rules for the design and manufacturing in fused deposition modeling (FDM) printing, we have experimentally selected both the material and the most important printing parameters. To do so, we have tested four commonly used and widely available polymer filaments: polylactide (PLA), polybutadiene-graft-poly(acrylonitrile-co-styrene) (ABS), polycarbonate (PC) and glycol-modified poly(ethylene terephthalate) (PET-G). Some of the physical properties of the filaments are presented in Table 1. PLA has the lowest temperatures of both: melting and glass transition. PET-G is significantly more resistant to high temperatures. Its softening temperature is 15 °C higher than that of the rest of the polymers. Polycarbonate combines high tensile and flexural modulus, but it requires printing at high temperatures. Values of the moduli of elasticity are comparable, except for PET-G which has much higher values. Among the materials used, ABS exhibited the highest susceptibility to thermal deformation, PC also struggles with thermal flex, while in PLA this problem was minimal.

**Figure 3:**
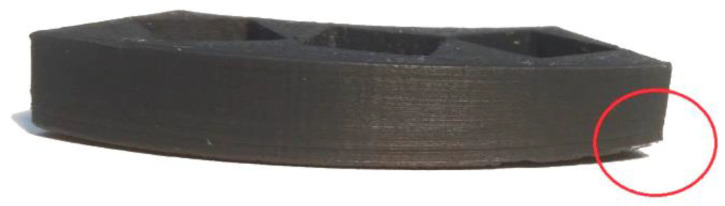
Warping in ABS printout circled in red.

Two main downsides of FDM printing are (i) thermal deformation and (ii) anisotropy of the printout. Deformations affect mainly the object’s outer layers, e.g., the corners or edges. The printing material left the nozzle at a temperature between 205–260 °C and then was cooled by air. The outer and middle layers, located away from both the heated nozzle and the heated platform, were most exposed to contraction. Occasionally, this leads the corners of the printouts to rise, as shown in Figure 3. This phenomenon is unfavorable not only due to the change in shape of the object, but it also can cause the printout to peel off the base, which, in extreme cases, may even destroy the printer [26].

To counteract this phenomenon, one has two possibilities. The first is to limit the air circulation by closing the printing chamber. The printer used in the project is semi-closed and only the upper part remains exposed. The second solution, also used in this project, was to increase the adhesion of the first layer by covering the glass platform with a special adhesive layer.

The anisotropy of the printed object is a consequence of the layered structure of the objects obtained with FDM. Connections between layers are always the weakest elements of the printout. The smallest force necessary to destroy the printout is therefore the tensile force, perpendicular to the layers. To ensure proper bounding of the layers, temperature of the nozzle, the base, and the cooling rate were set accordingly to the manufacturer’s recommendations listed in Table 2. Thermal settings are the key to good bounding, on the one hand the material must be in higher temperature long enough to fuse well with the previous layers, on the other the longer it stays hot, the more time it has to deform. In all cases, the layer height was 0.15 mm, which is standard for prints of this size.

During the main experiment, the rings were subjected to a series of forces due to the induced magnetic field, temperature, and their own weight, or CO_2_. These forces must under no circumstances deform the elements of the set, for fear of damaging the equipment and changing the properties of the magnetic field, thus destroying the experiment. Interacting magnets mounted on the polymer ring were the source of many forces and moments with vector directions difficult to define. Thus, it was difficult to determine which parameters—e.g., toughness, elasticity, infill, or glass transition temperature—were crucial for the final effect.

**Figure 4:**
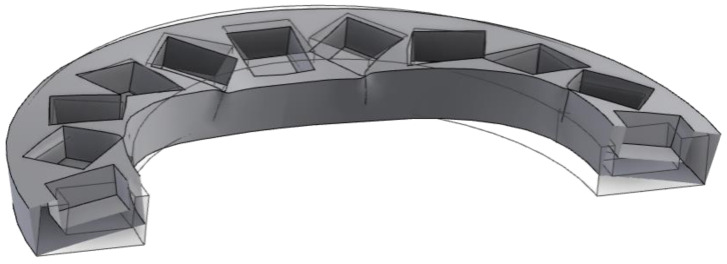
Schematic drawing indicating the changes of ring dimensions during the thermo-magnetic measurements.

An observation of the living cells must be conducted in the temperature of 37 °C. To test how the rings made from thermoplastic would respond to the strain caused by interacting magnets in higher temperatures, two stage test was conducted. Firstly, we tested the impact of filling, namely: 25, 50, 75, and 100% grid infill on the mechanical performance. PLA rings were printed and filled with twenty cubic magnets with an edge length of 10 mm and then placed in a cell incubator for 72 h at the temperature of 37 °C in the atmosphere simulating humidity and CO_2_ concentration such as during the biological measurements. Every 12 h, the printouts were removed from the incubator to determine their deformation. After that additional rings of PC, ABS, and PET-G were printed with the most durable infill and inserted with magnets. The strain generated by the repulsion of the magnets caused all PC rings to snap during inserting the magnets, which excluded this material from further tests. PLA, ABS, and PET-G rings were tested in the same manner. Deformation of the ring is schematically shown in Figure 4.

**Figure 5:**
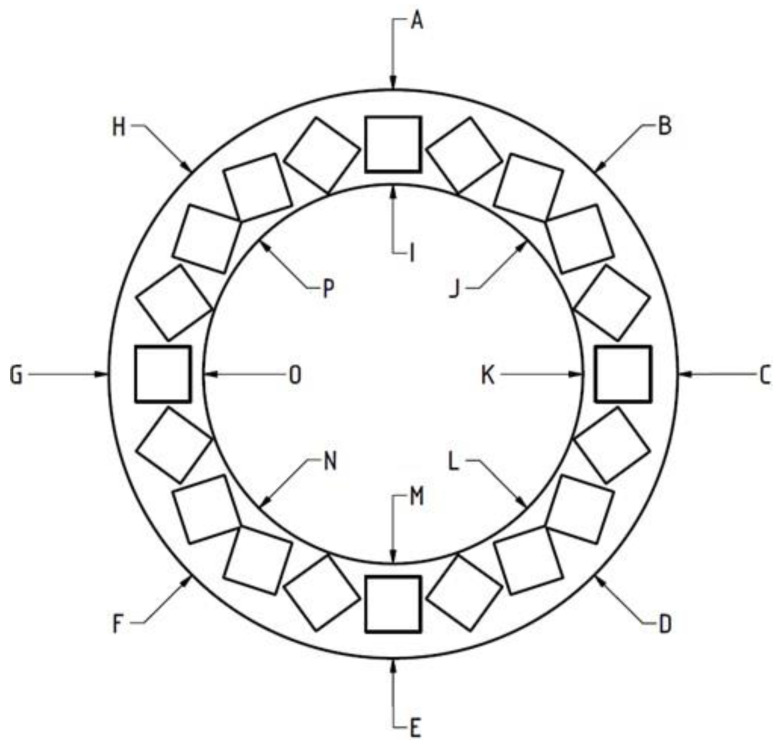
Ring used in the thermal tests with positions of points A–P (see text).

To measure the deformations, heights of the rings were measured at 16 distinct points marked in Figure 5. Table 3 and Table 4 contain the ranges of measured dimensions and their fractional change, expressed as the difference between the height after and before the incubation.

It was found that the inner side of the ring was subject to greater deformation than the outer one, leading to arching of the ring. The largest deformations were observed for rings with 75% filling, followed by those with 100% filling. In the case of 25% and 50% filling, the average deformations are much smaller (Table 3). Based on the results, 100, 75, and 25% fillings were rejected, and the 50% filling was selected for the final printouts.

Among all tested materials, ABS had the highest deformation under exposed conditions. PLA, although having the smallest fractional change of dimensions, was excluded due to the vast height range. Deformations in PLA were the most ununiform with the difference of over 1 mm between some of the points. After all tests, PET-G has been selected for printing the rings and mounting frames. It was easily printable, warping was minimal, and it was the most stable in the thermal deformation tests.

Magnetic induction inside the ring-shaped magnets was measured before incubation and after 72 h incubation. No changes in the magnetic field were observed for any of the rings.

### 3.3. The Halbach Magnets

**Figure 6:**
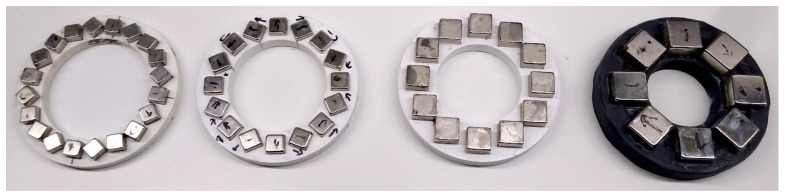
Halbach arrangements of 10, 12, 15, and 20 mm cubic magnets.

A properly constructed Halbach array produces a uniform gradient of the magnetic field, rising from the center of the ring to the edges. The appropriate permanent magnets and their arrangement were selected as described in Section 3.1. Four architectures were tested, in which 20-, 15-, 12- and 10 mm cubic neodymium magnets were used. The exemplary magnets are shown in Figure 6. They have been marked in accordance with the length of the side of the magnet in millimeters. Note that all magnetic rings have the same external diameter and thicknesses, so they easily fit the mounting frame.

The induction of the magnetic field generated by the array was measured inside a square located in the center of the ring, with a sensor mounted on a tripod on the optical stage. Each square was divided into smaller areas on a 5 mm grid. The length of the square’s sides was 40 mm for the ring with 10 mm magnet and 30 mm in the case of a ring with 20 mm magnet. The position of the sensor was controlled with a set of micrometer screws. Measurements were made in two mutually perpendicular directions, and the results were added according to the rules of vector calculus.

**Figure 7:**
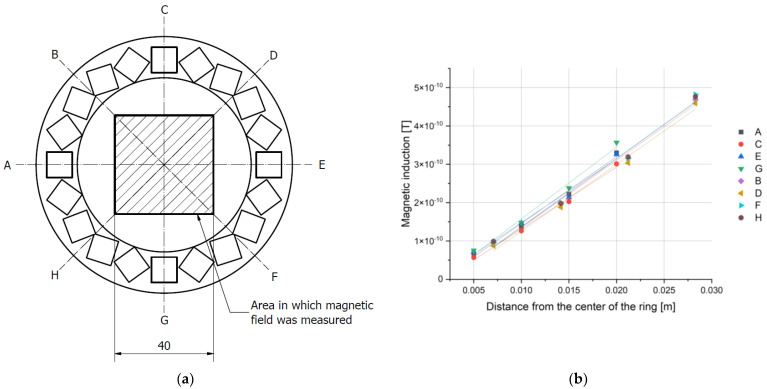
Magnetic field induction measurements for 10 mm magnets: (**a**) Schematic drawing of a ring used during the measurements with marked radii; (**b**) Magnetic field induction along the radii.

**Figure 8:**
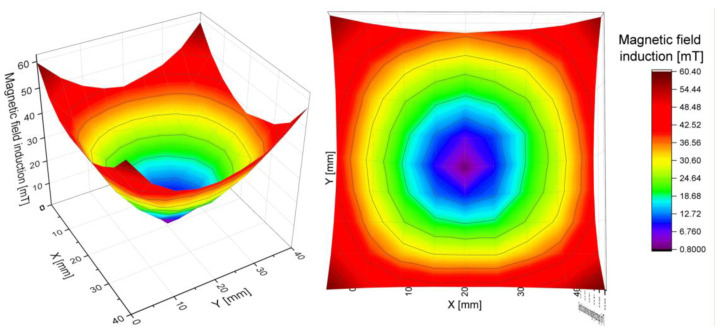
Visualisation of the magnetic field induction inside the ring with 10 mm magnets.

The results obtained in the XY-plane allowed for the calculation of the total induction at each point of the grid. As predicted, it was lowest in the center of the ring and increased radially, with an approximately constant gradient, specific for each array. By measuring the magnetic induction along 8 directions (Figure 7 and Figure 8), we have confirmed that the magnetic field gradient was the same in each of them.

The results were fitted with a simple linear regression (R^2^ > 0.99). Because the gradient is given by the tangent of the slope of the function, the values of the slopes of the fitted lines were compared. They were consistent for each ring within the limits of the measurement uncertainty. Thus, the gradient of the magnetic field for each ring was determined as the average value of the slope coefficient for all lines. The maximum uncertainty obtained from those measurements was assumed as the uncertainty of the final result. We conclude that the magnetic field inside each of the rings has a constant, uniform gradient (Figure 8 and Table 5).

The results confirmed that the magnetic field gradient was constant for each ring at each measured point. Since the system was planned for a specific set of biologically relevant experiments, the condition for values of magnetic induction, resulting in a high enough magnetic force, was found to be satisfied for the 20-35-08 and 10-85-20 rings, while for the 05-90-40 ring the magnetic induction was too low. Additionally, 10-85-20 ring was a source of almost 10 times stronger field than 20-35-08.

To adapt the setup to biological samples, it is crucial to add functionalities which provide and maintain the proper conditions, i.e., temperature, humidity, and the level of carbon dioxide. Temperature control was accomplished through an external heating chamber, available to the microscope and applied to its entire stage. Humidity and CO_2_ concentration were controlled by additional external microscope modules. An alternative solution would be to design a heating cap on the stand, but the solution was rejected due to the higher complexity and lower convenience of using such a structure.

### 3.4. Validation of the Halbach Magnet

To validate the magnitude of the magnetic forces generated in the Halbach magnet, it was tested in a series of simple experiments, where magnetic beads of known diameters were placed inside the array and their motion was recorded under the microscope. From the obtained trajectories of the beads, the drag force has been estimated. In short, for a spherical particle with radius R, moving with velocity v in a liquid having viscosity η, the drag force is given by:

$$F_{drag}=6\pi \eta Rv$$

Knowing that in the case of a motion with constant speed the drag is being balanced by a drag force, the latter can be easily found.

The beads used in the experiments were 500 nm in diameter. Their cores were made of iron oxide γ-Fe_2_O_3_ (maghemite). Initial aqueous bead suspensions of 50 mg/mL were diluted with distilled water (1 μL of beads per 1000 μL of water). The solution was then mixed by pipetting it 10 times and finally 750 μL of the suspension was placed in a glass microwell dish inside a Halbach array mounted on a printed frame and the original microscopic stage. To limit the influence of external magnetic fields, measurements were carried inside a Faraday cage.

**Figure 9:**
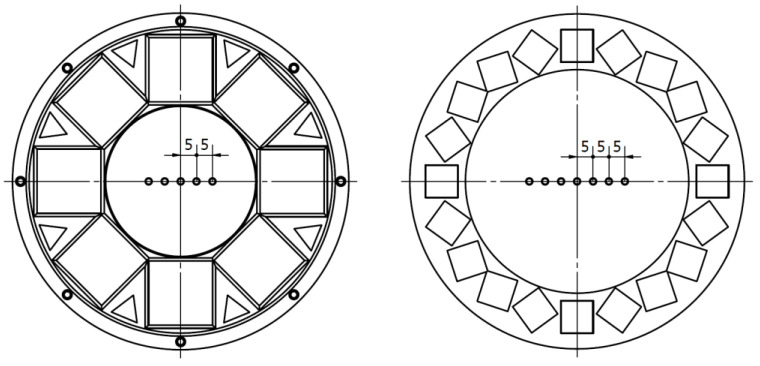
Measurement points for 20 mm (**left**) and 10 mm (**right**) magnet arrays.

Observations of the bead displacement were conducted for magnetic rings with 10- and 20 mm magnets (the smallest and the largest ones) along a selected diagonal at different distances from the center of the ring—see Figure 9. At each point, one hundred images of the moving spheres were taken at an interval of 200 milliseconds. Using the free ImageJ program with the Bio-Format plugin [27], images were converted into a movie. Using Tracker [28] program, the positions of the beads on each frame were determined and, by knowing the time interval between frames and the magnification of the lens, their speed was calculated. At least 15 beads were traced on every frame for at least 20 frames each. Obtained speeds follow the normal distribution, which has been confirmed with the Shapiro–Wilk test. The maximum of the peak of the normal distribution was assumed to be representative for the speed value at a given point. Speeds were further recalculated to obtain drag and magnetic forces. Obtained results are shown in Table 6. As expected, the magnetic force increased with the radius from the center of the ring. 

**Figure 10:**
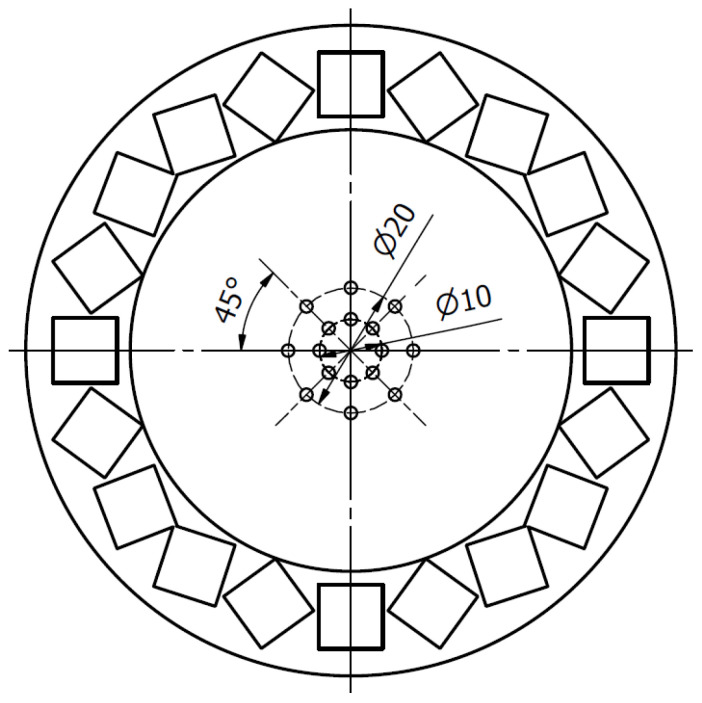
Measurement points in the second experiment—in the ring with 5 and 10 mm radii.

To test whether the drag (and therefore equal magnetic) force acting on the spheres is constant for a given value of magnetic induction—i.e., for a given distance from the center of the ring—we have calculated the drag forces acting on a moving bead 5 and 10 mm from the center of the array—see Figure 10. This corresponds to approximately 10 and 20 mT, respectively, for a 10 mm magnets ring. Beads on a 20 mm circle were moved with almost twice the speed of 10 mm from the center (the corresponding drag forces were 0.14 ± 0.003 pN and 0.079 ± 0.001 pN, respectively), as expected.

## 4. Discussion

We have designed, manufactured, and tested a microscope frame with a Halbach array to provide the possibility of conducting measurements of biological materials within the controlled magnetic force field without disturbing their physiology in any other way. The generated magnetic field should be within the range of several hundred mT (preferably not larger than 200 mT), which is considered neutral for major types of cells and organelles [29,30]. It has been shown that such a field by itself does not change morphological nor physiological features of different types of cells. We have not seen any deviations from control in a 48 h cell viability assays, where we have used MEF 3T3 cells (data not shown). However, such a field can be used together with other factors, for example magnetic nanoparticles, or the magnetic force may not be constant, to, e.g., impact cell migration or even guide the migrating organism in a desired direction [31,32,33].

Our goal was to generate forces up to dozens of piconewtons, since they have been reported to be sufficiently high to move even large cellular structures, such as nuclei. We have successfully created a magnetic field of a given characteristic, suitable for this kind of observations, in particular with a constant magnetic gradient that allows for controlling the value of the magnetic force, as well as the values of magnetic induction considered safe for living cells. The measured magnetic forces resulting from the influence of the developed field also remain within the assumed range.

The elaborated system can be easy to adapt for different microscopes, since the obtained field relies only on the geometry of the Halbach array and not on how the ring is mounted on the frame. The solution allowed for the use of easy-to-handle Halbach magnets with various dimensions using only one stand. Changing the Halbach magnets in the microscope system was very convenient.

According to the microscopic observations, a single magnetic bead is subject to a force of magnitude ranging from a few hundredths of a piconewton (for a ring with 20 mm magnets and 500 mm beads) to several thousandths of piconewtons (for a ring with 10 mm magnets). Halbach array made it possible to obtain the desired magnetic field without the need to use electromagnets, and as a consequence, it simplified the system.

The use of 3D printing allows for quick and cheap manufacturing of the equipment and its parts. The choice of the family of materials was also not accidental: polymers are magnetically neutral materials, which simplifies the magnetic system, as the only sources of the field are magnets. However, the use of FDM technology also carries challenges, resulting mainly from the nature of the used materials, thermoplasticity being one of them. In the proposed application, operating at elevated temperature and significant forces generated by magnets, it was associated with the need to perform thorough tests of the developed solution and select the appropriate material.

The selected material, i.e., the PET-G filament, made it possible to obtain objects with the required parameters, the most important of which were those related to work at the required temperature, humidity and CO_2_ concentration, and at the same time it turned out to be extremely easy to print, as it is characterized by low heat shrinkage and relatively low printing temperatures. The selection of the right material is essential, because the limitations resulting from the dimensions of the microscope stage, objective and the magnetic ring have forced the base of the frame to be very thin, measuring tenths of a millimeter. The division of the setup into two separate objects, a ring and a mounting frame, allows, if necessary, to easily adjust the forces acting on the tested object, by changing the magnetic ring to another one. The frame is compatible with all manufactured rings, which all have the same diameter and thickness.

It was decided to use a large heating chamber placed over the entire movable microscope stage with a dedicated system controlling the air composition in the chamber. An alternative, smaller chamber, which would only control the temperature around the frame would pose additional requirements for the frame and rings, which would make the setup less versatile and more complicated.

**Figure 11:**
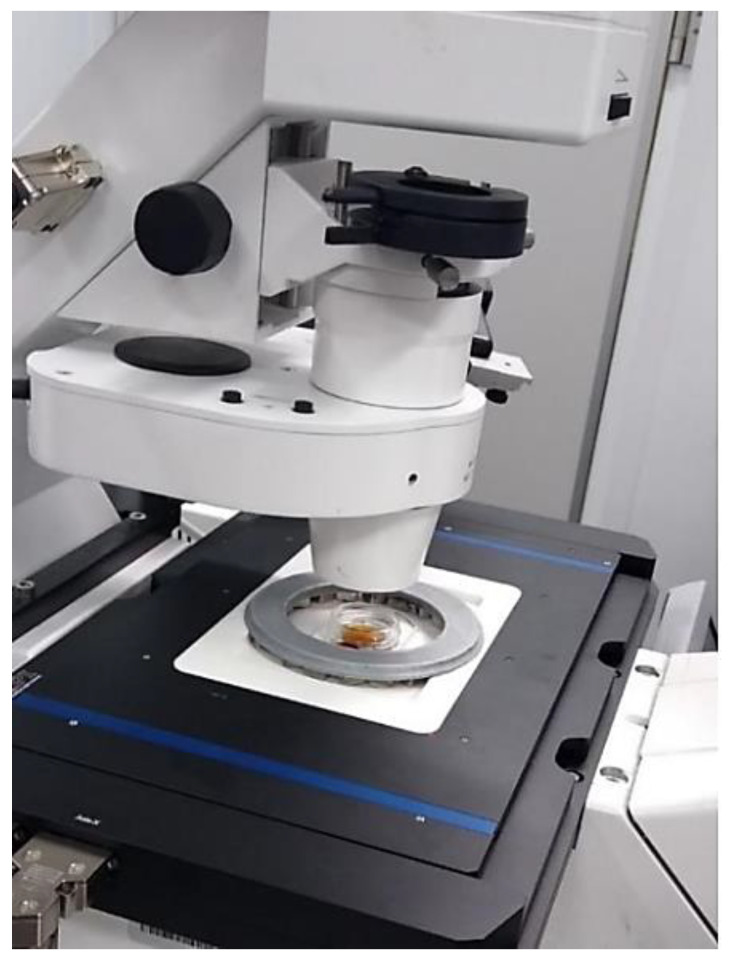
Mounting frame and the Halbach magnet mounted on the microscope.

Finally, the obtained structure, thanks to the use of a rounded rectangular hole, allows for microscopic observations along a 46 mm section of the ring diameter with 10 mm magnets. Since the values of the magnetic field generated by the ring are arranged radially, increasing diagonally from the center to the outside of the ring, the gradient of this change remains the same. This allows observations to be made with different force values at various points along this 46 mm line. At the same time, the radial arrangement of the magnetic induction values means that it depends only on the distance from the center of the ring, along any given diameter. Figure 11 shows the whole set.

We also note that, despite the fact that in a Halbach array magnets are arranged in a direct or quasi-direct repelling condition, i.e., they may demagnetize neighboring magnets, we have found no evidence of significant changes in the main characteristic of the magnetic field inside the ring, in the working area. For biological measurements, where the temperatures are limited by physiology and usually do not exceed 40 °C, this should not be an issue. It should be stressed that the proposed design is cheap and can easily be replaced after several uses. 3D printing the same model and using the same kind (manufacturer, size, etc.) of magnets reproduces a setup with the same properties, which is one of its advantages.

## 5. Conclusions

The paper presents an easily available method that enables biological measurements in the field of constant magnetic force. The approach is based on 3D-printed Halbach rings, in which different values of the gradient of the magnetic field, and thus the magnetic force, can be obtained by changing the magnets which are mounted on a stiff framework provided by the printout. Several common filaments for 3D printing were tested to find the most suitable for biological measurements, with the right structural durability and biological neutrality of printed elements. The future-oriented approach can be used in a variety of microscopic studies.

## Figures and Tables

**Table 1:** Properties of the filaments used for fabrication of frames.

	PLA	ABS	PC	PET-G
Glass temperature [°C]	62 ^1^		112–113 ^5^	80 ^6^
Softening point [°C]	63 ^1^	97 ^1^		78 ^1^
Melting point [°C]	151 ^5^	225–245 ^2^		
Tensile modulus [MPa]	1820 ^3^	1618.5 ^3^	2134 ^3^	3000 ^3^
Flexural modulus [MPa]	2490 ^4^	2070 ^4^	2410 ^4^	2040 ^4^

^1^ ISO 306; ^2^ ISO 294; ^3^ ISO 527; ^4^ ISO 178; ^5^ DSC 10 °C/min; ^6^ ASTM D3418.

**Table 2:** Printing parameters.

Polymer Filament	PLA	ABS	PC	PET-G
Nozzle temperature [°C]	205	250	260	225
Printer base temperature [°C]	50	80	110	65
Printing speed [mm/s]	80	60	50	60
Fan speed [%]	100	5	0	50

**Table 3:** Thermo-magnetic resistance test results of PLA rings depending on the filling of printouts.

Filling	25%	50%	75%	100%
Height range [mm]	8.00–9.40	8.30–9.30	8.70–10.40	8.48–10
Height change [%]	9.69	8.74	20.39	15.02

**Table 4:** Thermo-magnetic resistance test results of rings printed from different materials with 50% filling.

Material	PET-G	ABS	PLA
Height range [mm]	8.43–9.02	8.49–9.29	8.30–9.34
Height change [%]	9.29	10.86	9.20

**Table 5:** Measured magnetic field induction inside various rings.

Neodymium Magnets Dimensions	10	12	15	20
Magnetic field gradient [T/m]	2.18	2.5	3.75	20.46
Maximum induction in the area [mT]	60.40	155.56	176.92	526

**Table 6:** Magnetic force at different distances from the ring’s center.

10 mm Magnets		20 mm Magnets	
Distance [mm]	Force [pN]	Distance [mm]	Force [pN]
−15	0.18	-	-
−10	0.13	−10	0.42
−5	0.081	−5	0.20
+5	0.077	+5	0.21
+10	0.12	+10	0.42
+15	0.19	-	-

## References

[1] Rauch G., Sussman S., Hingorani N.G. (1991). Electric and magnetic fields: Background on health effects and an update on EPRI research. International Conference on AC and DC Power Transmission.

[2] Del Seppia C., Ghione S., Luschi P., Ossenkopp K.P., Choleris E., Kavaliers M. (2007). Pain perception and electromagnetic fields. Neurosci. Biobehav. Rev..

[3] Binhi V.N., Prato F.S. (2017). Biological effects of the hypomagnetic field: An analytical review of experiments and theories. PLoS ONE.

[4] Wachtel H., Beeman D., Pottenger J. Human responses to weak EMF are biologically plausible because “ordinary” electrically excitable channels can account for an extreme sensitivity to electric fields in sharks and related species. Proceedings of the 20th Annual International Conference of the IEEE Engineering in Medicine and Biology Society.

[5] Johnsen S., Lohmann K.J. (2008). Magnetoreception in animals. Phys. Today.

[6] Jeong J.H., Choi K.B., Yi B.C., Chun C.H., Sung K.Y., Sung J.Y. (2000). Effects of extremely low frequency magnetic fields on pain thresholds in mice: Roles of melatonin and opioids. J. Auton. Pharm..

[7] Matsunaga T., Tanaka T., Kisailus D. (2018). Biological Magnetic Materials and Applications.

[8] Savchenko V., Synyavskiy O., Nesvidimin A., Dudnyk A., Ramsh V., Bunko V. The impact of a direct magnetic field on the cells. Proceedings of the IEEE KhPI Week on Advanced Technology.

[9] Anderson L.E. Biological effects of magnetic fields: Laboratory studies. Proceedings of the 20th Annual International Conference of the IEEE Engineering in Medicine and Biology Society.

[10] Descamps L., Audry M.C., Howard J., Mekkaoui S., Albin C., Barthelemy D., Payen L., Garcia J., Laurenceau E., Le Roy D. (2021). Self-Assembled Permanent Micro-Magnets in a Polymer-Based Microfluidic Device for Magnetic Cell Sorting. Cells.

[11] Hashimoto Y., Kawasumi M., Saito M. (2007). Effect of Static Magnetic Field on Cell Migration. Electr. Eng. Jpn..

[12] Murray C., Pao E., Tseng P., Aftab S., Kulkarni R., Rettig M., Di Carlo D. (2016). Quantitative Magnetic Separation of Particles and Cells using Gradient Magnetic Ratcheting. Small.

[13] Ikeya N., Woodward J.R. (2021). Cellular autofluorescence is magnetic field sensitive. Proc. Natl. Acad. Sci. USA.

[14] Smid P., Shcherbakov V., Petersen N. (2015). Microscopic observation of magnetic bacteria in the magnetic field of a rotating permanent magnet. Rev. Sci. Instrum..

[15] Moisescu M.G., Leveque P., Bertrand J.R., Kovacs E., Mir L.M. (2008). Microscopic observation of living cells during their exposure to modulated electromagnetic fields. Bioelectrochemistry.

[16] Garanina A., Kireev I., Zhironkina O., Strelkova O., Shakhov A., Alieva I., Davydov V., Murugesan S., Khabashesku V., Majouga A. (2019). Long-term live cells observation of internalized fluorescent Fe@C nanoparticles in constant magnetic field. J. Nanobiotechnol..

[17] Lomelí-Mejía P.A., Tejeda-Buenosaires A., Alanís-Carbajal J. (2017). Construction of a Homogeneous Magnetic Field for Orthopedic Applications. Científica.

[18] Chou C.Y., Ferrage F., Aubert G., Sakellariou D. (2015). Simple method for the generation of multiple homogeneous field volumes inside the bore of superconducting magnets. Sci. Rep..

[19] Bednarek S., Płoszajski J. (2018). A coil generating high and homogenous magnetic fields. Bull. Soc. Sci. Lett. Łódź.

[20] Epherre R., Goglio G., Mornet S., Duguet E. (2011). 4.434—Hybrid Magnetic Nanoparticles for Targeted Delivery.

[21] Halbach K. (1980). Design of permanent multipole magnets with oriented rare earth cobalt material. Nucl. Inst. Meth..

[22] Halbach K. (1981). Physical and Optical Properties of Rare Earth Cobalt Magnets. Nucl. Inst. Meth. Phys. Res..

[23] Halbach K. (1985). Applications of Permanent Magnets in Accelerators and Electron Storage Rings. J. Appl. Phys..

[24] Doǧan N., Topkaya R., Subası H., Yerli Y., Rameev B. (2009). Development of Halbach magnet for potable NMR device. J. Phys. Conf. Ser..

[25] Sintered Neodymium-Iron-Boron Magnets. https://www.arnoldmagnetics.com/wp-content/uploads/2017/11/N42-151021.pdf.

[26] Ramian J., Ramian J., Dziob D. (2021). Thermal Deformations of Thermoplast during 3D Printing: Warping in the Case of ABS. Materials.

[27] Linkert M., Rueden C.T., Allan C., Burel J.-B., Moore W., Patterson A., Loranger B., Moore J., Neves C., MacDonald D. (2010). Metadata matters: Access to image data in the real world. J. Cell Biol..

[28] Brown D., Hanson R., Christian W. Computer Program Tracker Video Analysis and Modeling Tool, Version 6.0.2. 2022. https://physlets.org/tracker/.

[29] Miyakoshi J. (2016). The review of cellular effects of a static magnetic field. Sci. Technol. Adv. Mater..

[30] Zablotskii V., Polyakova T., Lunov O., Dejneka A. (2016). How a High-Gradient Magnetic Field Could Affect Cell Life. Sci. Rep..

[31] Dimonte A., Cifarelli A., Berzina T., Chiesi V., Ferro P., Besagni T., Albertini F., Adamatzky A., Erokhin V. (2015). Magnetic Nanoparticles-Loaded Physarum polycephalum: Directed Growth and Particles Distribution. Interdiscip. Sci. Comput. Life Sci..

[32] Blümler P. (2021). Magnetic Guiding with Permanent Magnets: Concept, Realization and Applications to Nanoparticles and Cells. Cells.

[33] Bongaerts M., Aizel K., Secret E., Jan A., Nahar T., Raudzus F., Neumann S., Telling N., Heumann R., Siaugue J.-M. (2020). Parallelized Manipulation of Adherent Living Cells by Magnetic Nanoparticles-Mediated Forces. Int. J. Mol. Sci..

